# Clinical performance of a new fissure sealant—results from a 2-year randomized clinical trial

**DOI:** 10.1007/s00784-022-04514-w

**Published:** 2022-05-02

**Authors:** Helen Schill, Peter Graeser, Katharina Bücher, Jan Pfisterer, Yeganeh Khazaei, Lukas Enggist, Reinhard Hickel, Jan Kühnisch

**Affiliations:** 1 Department of Conservative Dentistry and Periodontology, University Hospital, Ludwig-Maximilians-University Munich, Goethestraße 70, 80336 Munich, Germany; 2 Dental practice, Wädenswil, Switzerland; 3 Ivoclar Vivadent AG, Schaan, Liechtenstein

**Keywords:** Pit and fissure sealant, Caries prevention, Retention rate, Survival probability, Split-mouth design, RCT, Kaplan–Meier statistics

## Abstract

**Objectives:**

The aim of this randomized clinical trial (RCT) was to explore the clinical survival of a new, Bis-GMA-free pit and fissure sealant (Helioseal F Plus) in comparison to an established control material (Helioseal F).

**Material and methods:**

This in vivo study was designed as a prospective, 2-year, two-centre RCT with a split-mouth design. The initial study population consisted of 92 adolescents who were followed up 1 month (*N* = 89), 6 months (*N* = 88), 1 year (*N* = 85) and 2 years (*N* = 82) after sealant application. The attrition rate was 10.9% after 2 years. At each examination, the sealant retention and presence of caries were recorded. The statistical analysis included the calculation of Kaplan–Meier survival curves, log-rank tests and a Cox proportional hazard regression model.

**Results:**

No adverse events during the application or any of the follow-up visits were documented. The proportion of completely intact sealants and those with minimal loss was almost identical in both groups at 85.9% (Helioseal F Plus) and 86.5% Helioseal F) after 2 years of observation. The regression analysis revealed operator dependency; no significant differences were found between the materials, the study centres, the chosen isolation technique and patient age or sex.

**Conclusion:**

The newly developed sealant can be evaluated as at least equivalent in terms of survival and retention behaviour compared to the established control material.

**Clinical relevance:**

The new sealant can be recommended for clinical use. With respect to the material properties (Bis-GMA-free, less light polymerisation time and better thixotropic behaviour), it offers additional advantages with clinical relevance.

## Introduction

Pit and fissure sealing is a preventive measure that is applied to susceptible caries sites, notably in young permanent teeth, aimed at preventing new caries or arresting existing non-cavitated caries lesions [1]. The effectiveness of this procedure has been proven in several scientific studies, systematic review [2] and meta-analyses [3–5]. Sealant materials have become widely used and accepted in recent decades and are constantly undergoing improvements regarding material properties to develop even safer and more effective materials [6, 7]. For some years now, the monomer Bis-GMA, contained in many dental composites and sealants, has been the subject of discussion due to its potential contamination with trace residues of hormone-active bisphenol A [7–9]. Therefore, manufacturers have aimed to reduce risks, and recently, a Bis-GMA-free sealant material (Helioseal F Plus, Ivoclar Vivadent AG, Schaan, Liechtenstein) was developed and introduced to the dental market. Compared to the predecessor product (Helioseal F, Ivoclar Vivadent AG, Schaan, Liechtenstein), the new fissure sealant exhibits the following clinical advantages: improved flowability and handling due to thixotropic effects and a shorter polymerization time (10 versus 20 s). Therefore, the purpose of this study was to investigate the clinical performance of this new fissure sealing material. The null hypothesis was that there were no statistically significant differences between the test and control materials, especially in terms of retention behaviours.

## Material and methods

### Study design and ethical approval

This in vivo study was designed as a prospective, two-centre, randomized clinical trial (RCT) with a split-mouth design. The split-mouth design was chosen to compare the test sealant (Helioseal F Plus, Ivoclar Vivadent AG, Schaan, Liechtenstein) with the control material (Helioseal F, Ivoclar Vivadent AG, Schaan, Liechtenstein). The clinical investigations were conducted at the Department of Conservative Dentistry and Periodontology of the LMU Munich, Germany, and a dental practice in Wädenswil, Switzerland, between 2018 and 2021. The clinical trial received ethical approval from the corresponding ethical boards (Ludwig-Maximilians University of Munich: Project number 18–319; Cantonal Ethics Committee in Zurich: Basec-Number 2018–00,707). All dental examinations were performed in accordance with the ethical standards of the institutional research board and the modified Helsinki Declaration [8]. Written authorization was obtained from all participating adolescents and their legally designated representatives. The reporting propositions of the CONSORT guidelines for RCTs were applied [9, 10].

### Inclusion and exclusion criteria

Children and adolescents between 5 and 18 years of age were selected as the patient group, as they particularly benefit from this preventive measure and can thus be defined as the primary target group [1, 11, 12]. Following the recommendations of both the German and European guidelines, patients with an increased caries risk, healthy teeth but with fissures susceptible to caries and fissures with an initial lesion limited to the enamel were included [1, 12]. Additionally, patient-related inclusion criteria were the absence of allergies and a known intolerance to methacrylate or other ingredients of dental sealants or restorative materials. Furthermore, patients with severe medical restrictions (ASA status > 1) were excluded [13, 14], and all patients with restoration-requiring cavities or dentin lesions received treatment before inclusion.

The tooth-related inclusion criteria were the presence of at least one fully or partially unsealed pair of permanent first or second molars; these could either be caries-free or have a non-cavitated carious lesion. Cavitated molars with an ICDAS score > 3 [15] and teeth with hypomineralization or any other structural or shape aberrations were excluded from the study. Extensively restored teeth with small sealable areas on the occlusal surface were also excluded. Similarly, fissures or pits of deciduous teeth, premolars, permanent canines and anterior teeth were not included.

### Sample size calculation, patient screening and follow-ups

Before conducting the split-mouth study, a sample size calculation was performed using G-Power software version 3.1.9.7. [16]. Here, an alpha of 5%, a confidence interval of 95% and an effect size of 0.60 with two groups, each with at least 40 individuals, yielded a power of 0.80. The study population was recruited from the patient pool in each study centre according to the previously described criteria. If the inclusion criteria were met, legally designated representatives were informed about the study project by the respective dentists (LMU: JP, JK, KB; Wädenswil: PG) and offered voluntary participation. In the case of a positive decision for participation, inclusion in the study was agreed upon. All the necessary examinations, application of fissure sealants (LMU: JK, HS; Wädenswil: PG) and detailed instructions on preventive measures appropriate for the indication were given at a separate appointment. All study participants were invited to follow-up examinations after 1 month (interval of 7 to 28 days after sealant application), 6 months (± 4 weeks), 1 year (± 2 months) and 2 years (± 2 months). In the case of a missed recall appointment, the legally designated representatives were encouraged by telephone up to two times to attend the examination. If no examination was performed, the corresponding recall visit was considered “lost to follow-up.” However, the patient was not excluded from the study. A subject was only dropped if either the study participant or the legally designated representatives were no longer able to continue the examinations e.g. due to a change of residence, or no longer wishing to participate at all. The complete loss or partial loss of a sealant, with initial caries lesions and necessary re-sealing, also led to the drop-out of the tooth in question. It should be noted that all study participants received access to dental care measures, in line with the indication at each study time point, with the preventive goal of maintaining overall dental health. This always included individual and aetiology-based health information, detailed instructions on age-appropriate oral hygiene, regular fluoridation measures and possibly invasive therapy measures.

### Blinding and randomization

To ensure an independent assessment of sealant quality, blinding between the operator and evaluator was performed. This was consistently possible at LMU. In contrast, blinding was not possible in the dental practice, as only one dentist (PG) worked there during the study period. With the aim of enabling an independent sealant evaluation by the study team, all sealants were photographed at each examination. At LMU, a professional single reflex lens camera (a Nikon D7100 or D7200 with a Nikon Micro 105-mm lens; Nikon, Tokyo, Japan) and a Macro Flash EM-140 DG (Sigma, Rödermark, Germany) were used after tooth cleaning and drying. Molar teeth were photographed indirectly using intraoral mirrors (Reflect-Rhod, Hager & Werken, Duisburg, Germany), which were heated before positioning in the oral cavity to prevent condensation on the mirror surface. In contrast, the newly sealed teeth were photographed directly using an intraoral camera (SiroCam AF^+^, Dentsply Sirona GmbH, Bensheim, Deutschland) connected to the dental chair at the dental office. The randomization of the sealing material on each pair of molars was realized by means of sealed and consecutively numbered envelopes, which were opened by the operator shortly before clinical execution. The choice of material was neither noted nor communicated to the study team or patient. Thus, a double-blinded study design was achievable at the university-based practice, and a single-blinded study design was possible at the dental practice.

### Study materials

The test material (Helioseal F Plus, Ivoclar Vivadent AG, Schaan, Liechtenstein, LOT at LMU and at the practice: W96091) is a newly formulated methacrylate-based hydroxy-methyl methacrylate (HEMA) phosphate, aromatic aliphatic urethane-dimethacrylate (UDMA), white-pigmented (silicon dioxide, titanium oxide), fluoride-releasing (aluminium fluorosilicate glass) and light-curing (campherquinone with absorption at a 400-–500-nm wavelength) fissure sealant. The product is unique for several reasons. It is Bis-GMA-free, exhibits thixotropic behaviour, enhances flow into deep fissures and has a short polymerization time of 10 s. Helioseal F (Ivoclar Vivadent AG, Schaan, Liechtenstein, LOT at LMU and at the practice: X23069) was chosen as the control material. Its composition has been reported elsewhere [17].

### *Sealant application*

Trained dentists (LMU: JK & HS, Dental practice: PG) carefully conducted all clinical procedures using a professional dental unit with an operation light, a plane dental mirror and compressed air. At the beginning of each diagnostic examination, professional tooth cleaning was conducted. At LMU, all visible surfaces were cleaned with a medium abrasive fluoride-free polishing paste (Proxyt® Prophy Paste, RDA 36 Ivoclar Vivadent AG, Schaan, Liechtenstein, LOT: X19757) and a rotating bristle brush (Prophy Brush, Hager & Werken GmbH & Co KG, Duisburg, Germany). At the dental office in Wärdenswil, all teeth, especially those with fissures, were cleaned with a water-infused air powder polishing system (Prophy Mate Neo and Flash Pearl, NSK Europe, Bensheim, Germany). The application of both sealants was strictly performed according to the manufacturer’s recommendations. Therefore, after proper rinsing with water spray and drying with water-free and oil-free air, relative isolation with cotton rolls was used at the LMU study centre. In contrast, absolute isolation with a rubber dam (Dental Dam, Coltène, Altstätten, SG, Schweiz) and rubber dam clamps (Ivory, Kulzer, Wasserburg, Deutschland) was utilized at the dental practice. Afterwards, an etching procedure with 37% phosphoric acid gel (Total Etch®, Ivoclar Vivadent AG, Schaan, Liechtenstein, LOT: X23292) was performed for 60 s. The tooth surface was then rinsed with water spray for 5 s and air-dried for another 5 s until a chalky-white enamel surface was visible. A small amount of both materials was discarded in advance due to the possibility of air bubbles in the cannula body. The sealant was then applied to the fissure pattern and distributed with the cannula and dental probe (dental office) or brush stick (University: Microbrush®, Microbrush Int., Grafton, WI, USA) aiming to cover all pits and fissures completely without incorporating air bubbles. The test and control materials were exposed for 10 s (Helioseal F Plus) and for 20 s (Helioseal F) with an LED polymerization lamp with a light intensity of 1.200 mW/cm^2^ ± 10% (Bluephase Style®, Ivoclar Vivadent AG, Schaan, Liechtenstein), respectively. Furthermore, magnifying glasses were used when placing and checking the fissure sealant at the dental office. To remove the oxygen-inhibition layer, the bristle brush was used again. The occlusion was checked and, if necessary, adjusted with a polishing cup (OptraPol® Small Flame, Ivoclar Vivadent AG, Schaan, Liechtenstein). Finally, fluoride varnish was applied (University: Fluor Protector or Fluor Protector S, Ivoclar Vivadent AG, Schaan, Liechtenstein; Dental office: Elmex® fluid, CP GABA, Hamburg, Germany).

### Dental examinations and the calibration of the study team

The dental status included the recording of non-cavitated and cavitated caries lesions, as well as dental restorations, with respect to the established standard criteria [18–21]. Sealants and the extent of their retention were divided into the following categories [22–24]: 0 – occlusal surfaces without a sealant, 1 – occlusal surfaces with a fully intact fissure sealant (sufficient), 2 – an intact sealant with minor loss of the material up to one-third in the periphery of the fissure pattern (sufficient), 3 – occlusal surface with retention of the material in the main fissure but loss of the material exceeding one-third of the fissure pattern (insufficient) and 4 – almost a complete loss of the material and re-exposure of the main fissures (insufficient).

Prior to the study, a 2-day theoretical and practical calibration training, which focused on the clinical standardization of all examinations and test procedures, was conducted by a specialized dentist (JK) to instruct the other two operators (HS, PG) and examiners (KB, JP). The theoretical training exercise provided information regarding the study design, indices, diagnostic principles and standardized examinations and diagnostic procedures. Intra- and inte-rexaminer reproducibility was measured for all examiners and found to be sufficient. The weighted Kappa values for the intra- and inter-examiner reproducibility of the study team were good to excellent (ICDAS/UniViSS criteria: 0.90–0.97 (intra) and 0.89–0.97 (inter); DMF index: 0.85–0.86 (intra) and 0.76–0.92 (inter); sealant retention: 0.91–0.94 (intra) and 0.92–0.97 (inter)).

### *Statistical analysis*

Anonymous data collection was carried out by using a validated data entry and management system (“Evaluation,” Ivoclar Vivadent AG, Schaan, Liechtenstein). The anonymity of the data towards third parties and the sponsor was guaranteed, as access to the data was only possible for the principal investigator by entering an access code. After the export of the raw data, extensive plausibility checks were performed. Archiving of the anonymized data was performed at both study sites and on the sponsor’s secured servers. The data will be retained for 10 years. Descriptive analyses were conducted using Excel spreadsheets (Excel 2016, Version 16.53, Microsoft Corporation, Redmond, WA, USA). Explorative statistical analysis was performed using R software (Version R-4.1.1, R Development Core Team, Vienna, Austria). The significance level was set at *α* = 0.05 with a 95% confidence interval. Kaplan–Meier estimators were applied to generate data on the survival probability [25, 26]. Differences in the survival rate were assessed by applying log-rank tests. Cox proportional hazard regression analysis was carried out to investigate the influence of the variables of interest, such as age, sex, study centre, operator and material, on sealant survival after the 1-, 6-, 12- and 24-month follow-ups as well as a cumulative assessment over the whole study period.

## Results

**Fig. 1:**
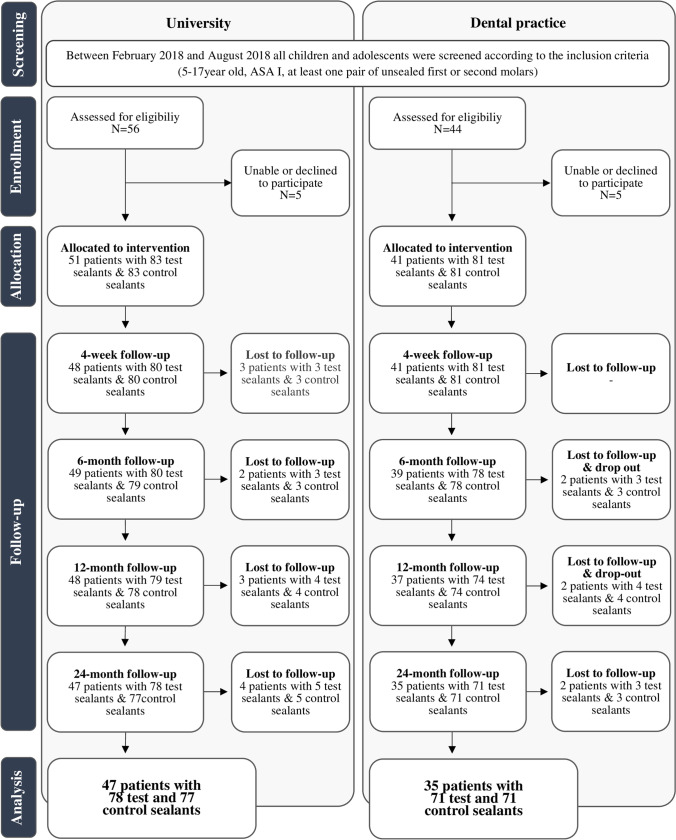
Study flow chart according to the CONSORT recommendations [9]

Of 92 individuals with a mean age of 9.6 years (standard deviation 2.9 years) who were initially included in the prospectively designed split-mouth study, 82 adolescents (mean age 11.7 years, standard deviation 2.8 years) could be followed up within the scheduled interval after two years (Fig. 1). This represents an attrition rate of 10.9%. Oral hygiene was documented as good in most of the subjects (Table 1). Furthermore, the caries experience of the study population was found to be low (Table 2).

**Table 1:** Characteristics of the study population

Study centre	University			Dental practice		
Visit	Baseline	1 year	2 years	Baseline	1 year	2 years
Number of individuals (N)	51	48	47	41	37	35
Age – mean (SD)	9.6 (2.7)	10.5 (2.8)	11.6 (2.8)	9.5 (2.7)	10.7 (2.8)	11.8 (2.8)
Female/male	25/26	25/23	25/22	26/15	22/15	20/15
Orthodontic treatment – no. of individuals with no/removable/fixed appliances	37/3/11	37/2/9	35/6/6	41/0/0	35/0/2	34/0/1
Oral hygiene in primary dentition – no. of individuals with no plaque/1–4 teeth with plaque/ > 4 teeth with plaque	34/9/8	30/13/5	37/9/1	31/2/8	21/5/11	25/2/8
Oral hygiene in primary dentition – Mean no. of teeth (sd) with plaque	2.0 (3.6)	1.3 (2.4)	0.6 (1.7)	1.7 (3.5)	2.9 (4.1)	2.3 (4.1)
Oral hygiene in permanent dentition – no. of individuals with no plaque/1–4 teeth with plaque/ > 4 teeth with plaque	27/13/11	16/23/9	19/13/15	17/5/19	12/7/18	17/4/14
Oral hygiene in permanent dentition – Mean no. of teeth (SD) with plaque	2.5 (3.5)	2.8 (3.0)	3.1 (3.6)	4.7 (4.4)	5.4 (5.3)	5.5 (6.3)
Periodontal health – no. of individuals with a PSI score of at least 0/1/2	27/10/14	15/3/30	8/9/30	18/20/3	24/10/3	22/10/3

**Table 2:** Caries experience of the study population

Study centre	University			Dental practice		
Visit	Baseline	1 year	2 years	Baseline	1 year	2 years
Caries experience in the primary dentition—mean (SD) dmf/t	**2.6 (1.9)**	**2.6 (1.7)**	**1.8 (1.2)**	**0.5 (0.9)**	**0.5 (1.0)**	**0.5 (1.0)**
d-Component (d/t)	0.0 (0.0)	0.2 (0.8)	0.1 (0.5)	0.0 (0.0)	0.0 (0.0)	0.0 (0.0)
m-Component (m/t)	0.3 (0.7)	0.1 (0.4)	0.1 (0.4)	0.0 (0.0)	0.0 (0.0)	0.0 (0.0)
f-Component (f/t)	2.3 (3.6)	2.3 (3.5)	1.6 (2.6)	0.5 (1.6)	0.5 (1.8)	0.5 (1.8)
Caries experience in the permanent dentition – mean (SD) DMF/T	**0.7 (0.9)**	**0.7 (0.8)**	**0.6 (1.1)**	**0.0 (0.1)**	**0.0 (0.0)**	**0.1 (0.4)**
D-Component (D/T)	0.0 (0.0)	0.0 (0.0)	0.0 (0.1)	0.0 (0.0)	0.0 (0.0)	0.0 (0.0)
M-Component (M/T)	0.0 (0.0)	0.1 (0.5)	0.0 (0.0)	0.0 (0.0)	0.0 (0.0)	0.0 (0.0)
F-Component (F/T)	0.7 (1.7)	0.6 (1.5)	0.6 (1.9)	0.0 (0.2)	0.0 (0.0)	0.1 (0.7)

In regard to the intended study aim, it can be reported that no material-related adverse events occurred during the application of the sealants or during follow-up visits.

Regarding sealant retention (Table 3), the following observations emerged in both study groups (Table 3). The proportion of completely intact sealants at the 24-month recall was almost identical in both groups, with proportions of 67.7% (Helioseal F Plus: 101/149) and 67.5% (Helioseal F: 100/148). Retention losses occurred equally in both material groups (Helioseal F Plus: 48/149, 32.2%; Helioseal F: 48/148, 32.4%). Most retention losses were classified as minimal (total: *N* = 55/297, 18.5%; Helioseal F Plus: 27/149, 18.1%; Helioseal F: 28/148, 18.9%). In contrast, a total of 41 sealants out of all the sealants (only 13.8%) were affected by more extensive retention loss central retention (*N* = 25), almost complete loss (*N* = 14) and complete loss (*N* = 2). These were found in roughly equal proportions in both material groups. Finally, the overall numbers of completely intact sealants and those with a minimal loss of materials (Helioseal F Plus: 85.9%; Helioseal F: 86.5%) were found to be high after 2 years of observation. In addition, the sealant margins were found to be intact in most cases (Table 3). Given the low number of events, the data were not explored further.

**Table 3:** Sealant retention in relation to the examination visits

	University									Dental practice								
	Test sealants				Control sealants					Test sealants					Control sealants			
**Sealant retention**	**1 month**	**6 months**	**1 year**	**2 years**	**1 month**	**6 months**	**1 year**	**2 years**	**1 month**		**6 month**	**1 year**	**2 years**	**1 month**		**6 months**	**1 year**	**2 years**
Intact sealant	74	66	51	41	72	64	49	44	81		67	65	60	81		68	62	56
Minimal loss of retention	6	4	12	18	8	7	13	16	-		9	9	9	-		10	11	12
Main retention complete	-	7	6	10	-	4	12	11	-		-	-	1	-		-	1	3
Nearly complete sealant loss	-	2	8	8	-	2	2	6	-		2	-	-	-		-	-	-
Complete sealant loss	-	1	2	1	-	2	2	-	-		-	-	1	-		-	-	-
Total	80	80	79	78	80	79	78	77	81		78	74	71	81		78	74	71
**Marginal integrity**	**1 month**	**6 months**	**1 year**	**2 years**	**1 month**	**6 months**	**1 year**	**2 years**	**1 month**		**6 months**	**1 year**	**2 years**	**1 month**		**6 months**	**1 year**	**2 years**
Sufficient, minimal leakage	80	80	78	78	80	78	77	76	81		78	74	71	81		77	74	71
Insufficient/partially mobile/lost	–	–	1	–	–	1	1	1	–		–	–	–	–		1	–	–
Total	80	80	79	78	80	79	78	77	81		78	74	71	81		78	74	71
**New caries lesions on sealed teeth**	**1 month**	**6 months**	**1 year**	**2 years**	**1 month**	**6 months**	**1 year**	**2 years**	**1 month**		**6 months**	**1 year**	**2 years**	**1 month**		**6 months**	**1 year**	**2 years**
No caries	80	80	77	78	80	79	77	76	81		78	74	71	80		78	74	71
Non-cavitated caries	–	–	2	–	–	–	1	1	–		–	–	–	1		–	–	–
Cavitated caries	–	–	–	–	–	–	–	–	–		–	–	–	–		–	–	–
Total	80	80	79	78	80	79	78	77	81		78	74	71	81		78	74	71

New non-cavitated caries lesions were documented in five permanent molars in five different study participants; no caries-related cavitations were diagnosed within the study interval (Table 3). When considering these numbers in relation to the overall sealed number of molars after 2 years (*N* = 297), the percentage of healthy occlusal surfaces amounted to 98.3% (threshold of non-cavitated caries lesions) and 100.0% (threshold cavitated caries lesions). Due to the low number of new caries lesions, it was not feasible to explore the data statistically.

**Fig. 2:**
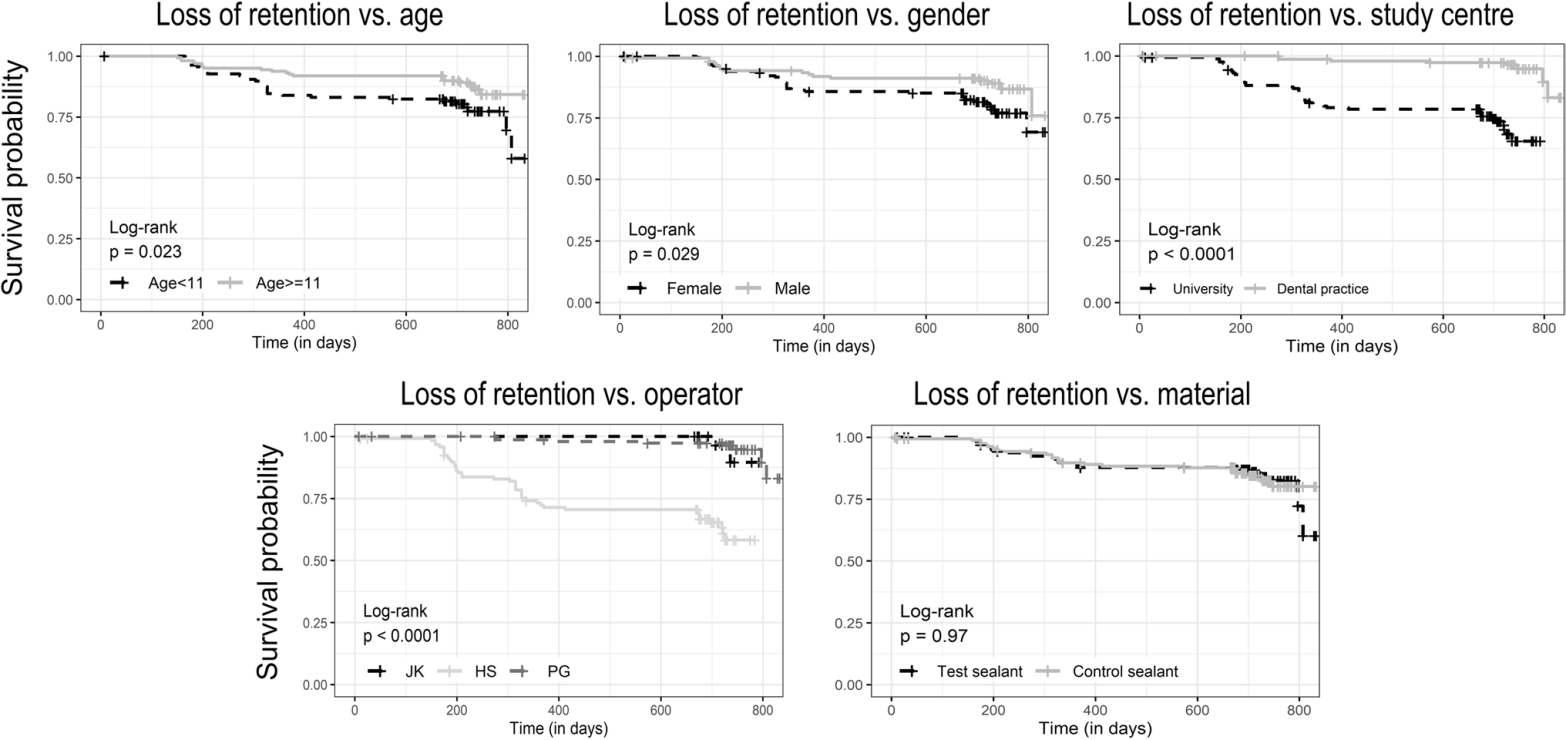
Kaplan–Meier survival curves illustrating the survival probability in relation to the chosen co-variables

The dataset was further explored by using a logistic regression model (Table 4) and by illustrating survival probabilities in relation to different variables by using Kaplan–Meier analysis (Fig. 2). In detail, the curves indicate age, sex, study centre and operator dependency after 2 years of observation by applying log-rank tests (Fig. 2). The Cox proportional hazard regression analysis under inclusion of the cumulative retention data as well as important co-variables from all follow-up examinations indicated only operator dependency (Table 4). Furthermore, no significant differences regarding age, sex, study centre or sealant material were detectable (Table 4).

**Table 4:** Results from the Cox hazard models used to analyze potential associations between sealant retention and relevant co-variants. In the first approach, only retention data from the 2-year visit were accessed; in the second model, retention data from all study time points were calculated

Loss of retention Hazard ratio (95% CI)	Retention after 1 month	Retention after 6 months	Retention after 1 year	Retention after 2 years	Cumulative retention
Age < 11 years	1	1	1	1	1
Age ≥ 11 years	1.43*e − 09 (0.00-Inf) *p* = 1.00	0.76 (0.28–2.02) *p* = 0.58	0.52 (0.25–1.12) *p* = 0.10	**0.46 (0.24–0.87)** ***p* = 0.02**	0.69 (0.39–1.25) *p* = 0.22
Sex – female	1	1	1	1	1
Sex – male	1.79*e + 09 (0.00-Inf) *p* = 1.00	1.23 (0.46–3.29) *p* = 0.68	0.64 (0.30–1.36) *p* = 0.25	**0.34 (0.17–0.67)** ***p* = 0.002**	0.55 (0.29–1.03) *p* = 0.06
Study centre – university	1	1	1	1	1
Study centre – dental practice	1.37 (0.00-Inf) *p* = 1.00	0.64 (0.00-Inf) *p* = 1.00	2.99*e + 06 (0.00-Inf) *p* = 0.99	0.23 (0.04–1.32) *p* = 0.10	0.60 (0.12–3.03) *p* = 0.54
Physician – JK & PG	1	1	1	1	1
Physician – HS	4.07*e + 09 (0.00-Inf) *p* = 1.00	1.51*e + 09 (0.00-Inf) *p* = 0.99	2.33*e + 08 (0.00-Inf) *p* = 0.99	**6.29 (1.49–26.58)** ***p* = 0.01**	**7.71 (1.82–32.75)** ***p* = 0.005**
Test sealants	1	1	1	1	1
Control sealants	1.61*e + 09 (0.00-Inf) *p* = 1.00	0.67 (0.27–1.65) *p* = 0.39	0.95 (0.49–1.84) *p* = 0.87	1.06 (0.61–1.86) *p* = 0.83	0.96 (0.55–1.66) *p* = 0.88

## Discussion

According to the given null hypothesis for this RCT, it was confirmed that no difference in terms of the retention behaviour was observed between the test and control materials. This seems to be of clinical importance because the new Bis-GMA-free material performs similarly to the control material in the clinical setting but features a lower toxicity and additional beneficial material properties, such as a 10 s polymerization time and improved thixotropy. Therefore, it might be considered more advantageous in daily routines.

The retention rates in terms of sufficiently sealed occlusal surfaces (Helioseal F Plus: 85.9%; Helioseal F: 86.5%; Table 3) are in line with the estimated expectations from previously conducted clinical trials and meta-analyses [3, 4] and fit into the known retention behaviour from past studies. As a rule of thumb, 80% of sufficiently sealed occlusal surfaces could be expected after 2 years of clinical observation. No material dependency was observed between the two materials (Table 4 and Fig. 2), which led to the conclusion that no superiority of one sealant was detectable.

The present RCT provides additional interesting details. When considering age as a potentially influencing variable, it was illustrated that slightly more favourable retention rates were found in older children than in younger children (Fig. 2). In contrast, observations from the Kaplan–Meier survival curves registered no significant influence when applying the Cox hazard regression model (Table 4).

It was possible to measure the influence of the isolation technique, which was used differently at both study centres (LMU: cotton rolls; dental practice: rubber dam). The corresponding Kaplan–Meier curve may indicate a difference, but this was found to be non-significant in the Cox hazard regression model. Therefore, it might be suggested that the choice of isolation had no obvious influence on sealant retention in our study [27, 28]. Nevertheless, there are also a few previously published reports that indicated a positive effect of absolute isolation with rubber dams regarding sealant retention [1, 29].

In regard to the detailed statistical analysis, it was found that retention losses were mostly attributable to one physician or their respective treated individuals (Table 4 and Fig. 2). Potential reasons could be less clinical experience (5 years) in treating children for this practitioner in comparison to the other two practitioners (> 20 years; with one of the practitioners specializing in paediatric dentistry), the challenging clinical management of young children and the familiarity of the whole study team. This might be an interesting side finding from the present RCT, which became observable within the Cox regression model but has been explored in only a minority of sealant studies (Table 4, Fig. 2). In this context, the differences between the study centres should be pointed out in more detail. As a specialized unit for children, which probably sees children with severe dental problems more frequently, the proportion of cooperation-demanding patients might also be higher at the LMU centre in comparison to the dental practice. This fact may result in a higher proportion of more challenging cases. Additionally, it should be noted that there was no further recording of the cooperation or compliance ability of the children, which might be discussed as a potential limitation of this study. The abovementioned fact might challenge younger physicians and may also explain the reduced retention rates at the university-based study centre.

The effect of caries prevention was determined as the second important variable. In teeth with sealant loss, non-cavitated carious lesions occurred on only 5 molars. No cavitations were detected, which would have required further operative interventions. The newly developed non-cavitated carious lesions were re-sealed, and a tooth-specific drop-out from the study was performed.

This study has strengths and limitations. A clear strength is the RCT design of the study and that it follows the CONSORT reporting recommendations. These existing standards allowed for direct comparison to derive causal interferences [10, 30]. The differentiation into age groups as well as the detailed recording of co-variables indicated potential influences on the sealing material. This allowed for a more detailed analysis (Table 4, Fig. 2), which in the present case also narrowed down the errors. The pandemic situation complicated the follow-up examinations and due to a change in residence or other accessibility issues, some study participants could no longer be followed up in both centres. Photographs were taken at each time point to maintain a patient-independent re-evaluation, allowing questionable cases to be discussed later with the principal investigator or by the study group. Nevertheless, the difference in the type of blinding between the two study centres should be mentioned. Since the practitioner in the dental practice had to evaluate his own applied fissure sealants, the collected data might be potentially biassed. Furthermore, it should not go unmentioned that there were differences in the preparation before applying the fissure sealant. The usage of a water-infused air powder polishing system might have beneficially affected the retention by enhancing the enamel surface with the resin compound, although clinical studies revealed no significant influence on retention [31–33]. Another possible limiting factor worth mentioning might be the study populations’ wide age spectrum (6 to 18 years) and the developing and varying occlusion pattern. Even though emphasis was placed on fine and precise application on fully erupted molars and occlusion checks just after placement, it cannot be eliminated whether a sealant was lost prematurely due to occlusal contacts. In addition, no data on patient cooperation or patient behaviour were collected at either study centre. This could be interesting for future studies and RCTs. Since only 2 years of data are available thus far, it is not yet possible to conclusively evaluate how the retention behaviour of the new sealant will compare in the long run. Long-term data are useful, as the influence of co-variables and their significance can only be determined after a longer observation period (Table 4).

## Conclusions

The newly developed material can be evaluated as at least equivalent to an established control material, with better properties (Bis-GMA-free, less light polymeristion time and better thixotropic behaviour) in this RCT and its evaluation. Additionally, further data should be explored for a long-term evaluation.
